# Impact of an Artificial Albumin Corona on Surface Charge-Driven Nano–Bio Interactions and Cytotoxicity of Silver Nanoparticles

**DOI:** 10.1021/acsomega.6c02733

**Published:** 2026-06-15

**Authors:** Marianna Barbalinardo, Emilia Benvenuti, Luana Mariani, Andrea Migliori, Lisa Lungaro, Giacomo Caio, Denis Gentili

**Affiliations:** † Consiglio Nazionale delle Ricerche, 9327 Istituto per lo Studio dei Materiali Nanostrutturati (CNR-ISMN), Via P. Gobetti 101, Bologna 40129, Italy; ‡ Consiglio Nazionale delle Ricerche, 201838 Istituto per la Sintesi Organica e la Fotoreattività (CNR-ISOF), Via P. Gobetti 101, Bologna 40129, Italy; § Department of Translational Medicine, St. Anna Hospital, University of Ferrara, Ferrara 44124, Italy

## Abstract

The clinical translation of nanomedicine remains limited by the low and often unpredictable delivery efficiency of nanoparticles, largely arising from protein corona formation and its complex interplay with nanoparticle surface properties. In this context, strategies based on engineered protein coronas have emerged to improve control over nano–bio interactions. In this work, we investigate the interplay between an artificial protein corona formed using bovine serum albumin (BSA), the most abundant serum protein, and surface charge, one of the key determinants of interactions at the nano–bio interface, in shaping the biological behavior of silver nanoparticles (AgNPs). Negatively charged citrate-stabilized AgNPs and positively charged PAH-coated AgNPs were precoated with BSA and evaluated across three cell lines selected for their different sensitivities to nanoparticle surface charge. Our results show that BSA precoating markedly enhances nanoparticle stability in complex media, particularly for positively charged AgNPs, yet does not significantly alter the qualitative composition of the adsorbed protein corona or the cytotoxic responses elicited by the nanoparticles. Under serum-free conditions, however, BSA coating becomes essential for maintaining colloidal stability and enabling cellular interactions, thereby allowing AgNPs to exert their intrinsic biological activity. These findings demonstrate that although the artificial corona provides important colloidal stabilization, surface charge remains the dominant factor governing the biological behavior of AgNPs, further highlighting the need to elucidate the mechanisms underlying charge-dependent interactions at the nano–bio interface for the rational engineering of nanoparticle systems.

## Graphical abstract

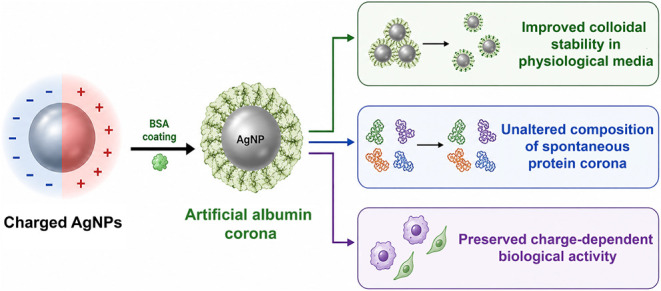

## 1 Introduction

Silver nanoparticles (AgNPs) are widely employed in diverse fields, including medicine, electronics, and the food industry, owing to their unique physicochemical properties. They exhibit antibacterial, antifungal, and anti-inflammatory activities, making them promising candidates for biomedical applications such as drug delivery and diagnostics.^1–3^ However, when exposed to biological environments, pristine nanoparticles are rapidly coated by biomolecules, particularly proteins, leading to the formation of the so-called protein corona on their surface. The protein corona strongly influences the physicochemical properties of NPs and significantly affects their biodistribution, cellular uptake, and cytotoxicity.^4–8^ It is well established that protein corona formation and composition are strongly influenced by both the surface properties of nanoparticles (charge, shape, size, and chemical composition) and the environmental conditions under which protein–nanoparticle interactions occur (temperature, pH, biomolecule concentration, and composition of the biological milieu). However, the adsorption of biomolecules onto nanoparticles remains a largely unpredictable and uncontrollable process, often regarded as an obstacle to their biomedical application,^9^ and has driven the development of strategies aimed at minimizing protein corona formation.^10^ At the same time, growing interest has focused on exploiting this phenomenon to deliberately engineer nanoparticles through the formation of an artificial corona to control interactions at the nano–bio interface and improve targeting and circulation properties.^11–15^ For example, Simon et al. precoated polymeric nanoparticles with immunoglobulin-depleted plasma to design a corona that reduced macrophage uptake.^16^ Similarly, Mirshafiee et al. demonstrated that a preformed artificial corona of γ-globulin on silica nanoparticles resulted in a protein corona enriched in immunoglobulins.^17^ Among serum proteins, albumin, one of the most abundant proteins in blood, has attracted considerable attention, and several studies have reported the coating of nanoparticles with bovine serum albumin (BSA) or human serum albumin (HSA). Albumin is generally classified as a dysopsonin, that is, a biomolecule whose adsorption onto the nanoparticle surface reduces cellular internalization.^18,19^ In agreement with this, several studies have reported that precoating nanoparticles with albumin typically reduces cellular uptake,^20–25^ but also the opposite effect has been observed, with albumin precoating enhancing nanoparticle internalization.^26–30^ Therefore, the extent to which precoating alters subsequent biomolecular corona formation and downstream biological responses remains insufficiently understood. In addition, we have recently reported that, even though the spontaneous formation of the protein corona in biological media masks the nanoparticle surface, the surface charge of AgNPs still strongly influences cellular interactions and intracellular behavior.^31^ These observations make it particularly relevant to understand whether the formation of a precorona can instead modulate, attenuate, or even override the charge-dependent biological effects of AgNPs.

Here, we report the effects of an artificial protein corona on the nanobio interactions and cytotoxicity of surface-charged AgNPs. Given the high abundance of albumin in biological fluids and its central yet sometimes contradictory role in modulating nanoparticle–cell interactions, we investigate how BSA precoating affects the physicochemical properties, subsequent protein adsorption, and biological activity of negatively charged citrate-stabilized AgNPs and positively charged poly­(allylamine hydrochloride) (PAH)-coated AgNPs. Through comprehensive physicochemical characterization combined with cytotoxicity assays in three cell lines, human breast adenocarcinoma (MCF-7), human colorectal adenocarcinoma (HT-29), and murine fibroblasts (NIH-3T3), we evaluate whether an artificial albumin corona can modulate (i) colloidal stability in complex biological media, (ii) the qualitative and quantitative composition of the serum protein corona, and (iii) nanoparticle-induced cytotoxicity. The three cell lines were selected to span a broad spectrum of charge-dependent sensitivities to AgNPs. NIH-3T3 fibroblasts respond to both cationic and anionic nanoparticles, MCF-7 cells are predominantly affected by positively charged AgNPs, and HT-29 cells remain largely unresponsive irrespective of surface charge.^31,32^ This deliberately chosen diversity in cellular sensitivity profiles is leveraged to evaluate whether BSA precoating exerts consistent modulatory effects on AgNP bioactivity across biological models with markedly distinct charge-response relationships. Complementarily, by assessing nanoparticle behavior under serum-free conditions, where spontaneous protein corona formation is precluded, we delineate the intrinsic biological impact of the engineered albumin layer.

Overall, our findings indicate that BSA precoating does not substantially alter either the composition of the adsorbed protein corona or the cytotoxic activity of the nanoparticles, regardless of surface charge, further supporting the predominant role of surface charge over protein corona composition in determining biological outcomes. Interestingly, under serum-free conditions, our data suggest that spontaneous protein adsorption onto charged AgNPs plays an essential role in colloidal stabilization in physiological environments, thereby enabling nanoparticle–cell interactions and the expression of their intrinsic biological activity.

## 2 Results and Discussion

### 2.1 Physicochemical Characterization

Negatively charged citrate-coated and positively charged PAH-coated AgNPs were synthesized following established protocols^31,33^ and subsequently coated with BSA, as detailed in the Experimental Section (see Figure 1a), to achieve AgNPs-cit@BSA and AgNPs-PAH@BSA, respectively. To prevent any potential effects arising from incomplete BSA coverage of the nanoparticles, the coating procedure was performed using BSA concentrations in large excess with respect to the nanoparticle surface requirements, as confirmed by BCA assay performed on the supernatant collected during the purification steps. The coating of negatively charged AgNPs with BSA was carried out in phosphate buffer at pH 7.4. However, under these conditions, the addition of BSA to positively AgNPs resulted in the formation of aggregates. This aggregation likely occurs because BSA carries a net negative charge at physiological pH and, as a result, can act as an electrostatic bridge between the NPs.^34^ To prevent this, the AgNPs-PAH were coated while maintaining the pH below the isoelectric point of BSA, ensuring that its net charge remained positive. As summarized in Figure 1a, the hydrodynamic diameter of the NPs increases upon BSA coating. Specifically, AgNPs-cit and AgNPs-PAH exhibit size increases of approximately 5 and 7 nm, respectively, suggesting that the adsorbed BSA is no longer in its native conformation. Similarly, the ζ-potential, which reflects the surface charge of the NPs, shows significant changes after BSA coating when measured at pH 7.4. For citrate-coated AgNPs, the ζ-potential becomes less negative, shifting from −38 mV to −29 mV. In contrast, for PAH-coated AgNPs, the ζ-potential reverses from a positive +39 mV to a negative −30 mV. Notably, when measured at a pH lower than the pI of BSA, the ζ-potential of both AgNPs-cit@BSA and AgNPs-PAH@BSA becomes positive (Figure 1a, values in brackets). The successful coating of the NPs with BSA was further confirmed by transmission electron microscopy (TEM). The as-synthesized AgNPs exhibit an average core diameter of 19 ± 2 nm, as determined by TEM. As shown in Figure 1b, the uniformly sized dark cores of the AgNPs are surrounded by a lighter, but distinct shell, indicating a consistent BSA coating. Furthermore, the characteristic absorption band at ∼400 nm, due to the localized surface plasmon resonance of AgNPs, undergoes only minor changes upon BSA coating for both AgNPs-cit and AgNPs-PAH. These changes include slight peak broadening and a minor red shift in the citrate-coated AgNPs, likely due to variations in the surrounding refractive index (Figure 1c). The minimal alterations in the absorption profiles suggest that the NPs largely remain nonaggregated after BSA coating.

**Figure 1:**
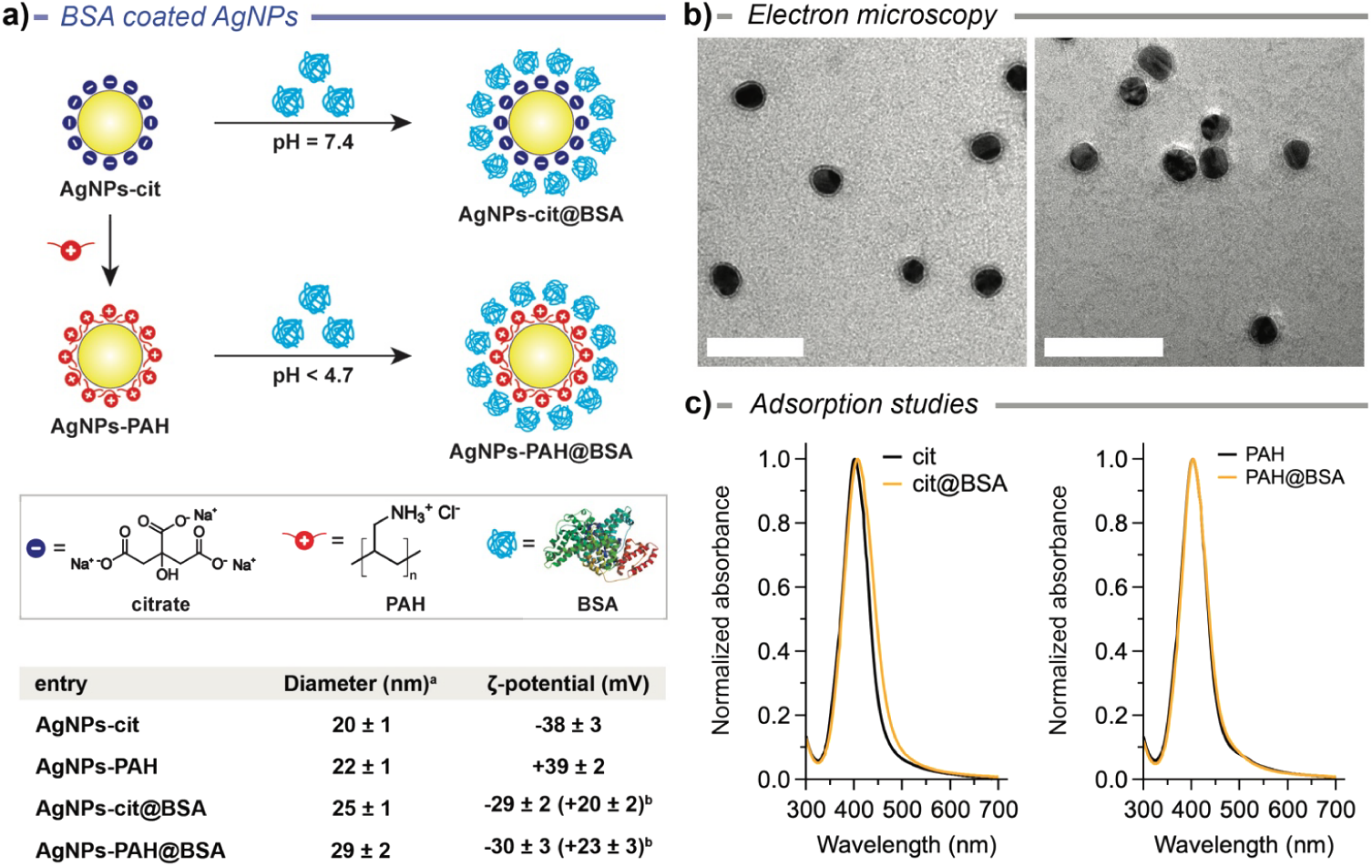
(a) Schematic representation of the various stages in the synthesis of AgNPs-cit@BSA and AgNPs-PAH@BSA, along with a table summarizing the physicochemical properties (^a^hydrodynamic diameter; ^b^measured at pH < pI of BSA). (b) TEM image of (left) AgNPs-cit@BSA and (right) AgNPs-PAH@BSA stained with phosphotungstic acid (see Experimental Section), scale bar: 100 nm. (c) UV/vis absorption spectra of (left) AgNPs-cit and AgNPs-cit@BSA, and (right) AgNPs-PAH and AgNPs-PAH@BSA.

### 2.2 Effect of BSA Precoating on Cytotoxicity of Nanoparticles

We evaluated the cytotoxicity of AgNPs-cit and AgNPs-PAH, both in their native form and with a precoating of BSA, on three different cell lines. Specifically, we tested two human tumor cell lines, MCF-7 (breast cancer) and HT-29 (colorectal cancer), as well as the murine fibroblast cell line NIH-3T3.

Positively charged nanoparticles (i.e., AgNPs-PAH) exhibit cytotoxic effects in MCF-7 cells (Figure 2a) and cause a slight reduction in cell viability in HT-29 cells (Figure S1), whereas no cytotoxic effects were observed in either cell line when treated with negatively charged AgNPs-cit, in agreement with previous reports.^31,32^ On the other hand, fibroblast viability drastically decreases after 48 h of exposure even to the negatively charged AgNPs-cit (Figure 2b). In MCF-7 and HT-29 cells, BSA precoating does not significantly affect the cytotoxicity of AgNPs-PAH, as AgNPs-PAH@BSA exhibits a similar effect at both 24 and 48 h. In contrast, in NIH-3T3 fibroblasts, BSA precoating slightly mitigates the cytotoxicity of AgNPs after 48 h, particularly for positively charged nanoparticles, compared to their uncoated counterparts, although this mitigating effect remains modest and cytotoxicity is still significant even in the presence of a BSA precorona. As AgNPs must be internalized to exert their toxic activity,^35^ these results suggest that cellular uptake remains largely unaffected by BSA precoating in MCF-7 and HT-29 cells, whereas it is likely modestly reduced in NIH-3T3 fibroblasts, where BSA precoating is associated with a moderate yet incomplete attenuation of the cytotoxic response.

**Figure 2:**
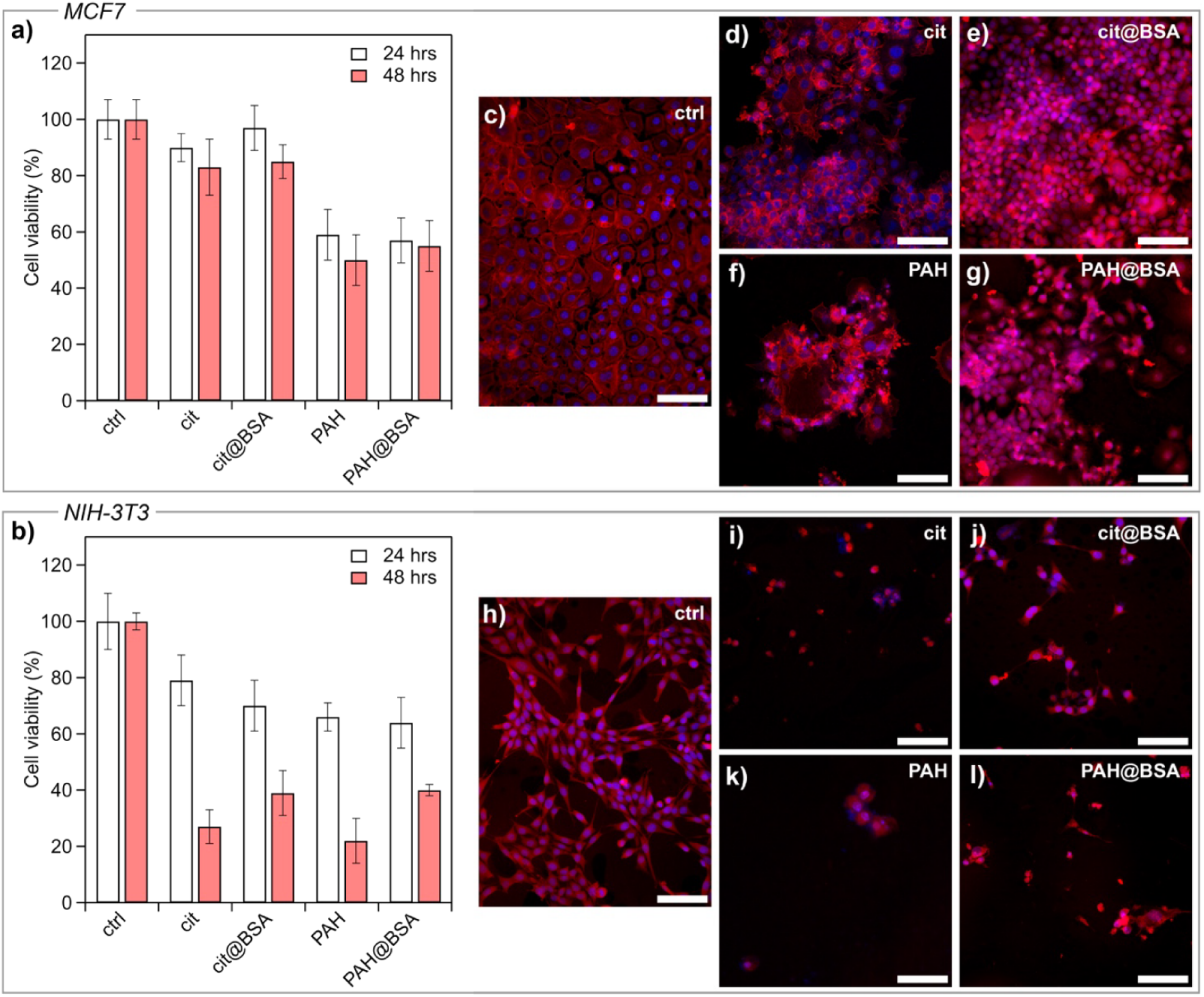
Cell viability of (a) MCF-7 and (b) NIH-3T3 cells treated for 24 and 48 h with AgNPs (20 μg/mL) as a function of surface coating. Data represent the mean ± SD and are expressed as a percentage relative to control samples (ctrl). At least four independent experiments were conducted. Fluorescence micrographs of (c–g) MCF-7 and (h-l) NIH-3T3 cells specifically labeled for actin (red) and nuclei (blue), treated for 48 h with AgNPs-cit, AgNPs-cit@BSA, AgNPs-PAH, AgNPs-PAH@BSA (20 μg/mL) and with vehicle solution (ctrl). Scale bar: 100 μm.

### 2.3 Effect on Cell Morphology

Figures 2d–g and S2 show fluorescence micrographs of MCF-7, NIH-3T3 and HT-29 cells stained for actin (red) and nuclei (blue) after 48 h of treatment with AgNPs. Following treatment with AgNPs-cit and AgNPs-cit@BSA (Figure 2d-e), MCF-7 cells largely retain their morphology, closely resembling those treated with the vehicle solution (i.e., the control; Figure 2c), with preserved actin structure and nuclear integrity, suggesting minimal cytotoxicity. In sharp contrast, cells treated with AgNPs-PAH and AgNPs-PAH@BSA (Figure 2f–g) exhibit marked disruption of the actin cytoskeleton and a visible reduction in cell density, revealing the cytotoxic effects of the nanoparticles, consistent with the viability data in Figure 2a. In NIH-3T3 cells, all treatments (Figure 2i–l) lead to a drastic decrease in cell density, and the typical elongated, spindle-shaped morphology of NIH-3T3 cells seen in the control (Figure 2h) is impaired, with cells displaying retracted, rounded shapes and compromised actin architecture. These results highlight the cytotoxic effect of AgNPs on NIH-3T3 cells regardless of surface coating and align with the observed decrease in cell viability (Figure 2b). Fluorescence microscopy images (Figure S2) reveal that HT-29 cells treated with AgNPs-cit and AgNPs-cit@BSA maintain a relatively intact morphology, with well-preserved actin filaments and defined nuclei, comparable to the control. On the other hand, exposure to AgNPs-PAH and AgNPs-PAH@BSA does not result in a drastic reduction in cell number, but clear cytoskeletal disruption and loss of structural integrity are observed. These qualitative differences in cell morphology are consistent with the cell viability data (Figure S1).

### 2.4 Effect of BSA Precoating on Protein Corona

The adsorption of proteins onto the surface of AgNPs, leading to the formation of the protein corona, is a crucial step in determining the interaction of nanoparticles with cells and, consequently, in defining their cytotoxic activity.^33,35^ Therefore, to assess the effect of precoating with BSA on protein corona formation, we exposed the AgNPs, with and without BSA precoating, to serum protein-containing cell growth media used for the fibroblast cell line (NIH-3T3) and for human cancer cell lines (MCF-7 and HT-29). The time evolution of the resulting NP-protein complexes was monitored by UV–vis spectroscopy and dynamic light scattering.

Figures 3 and S3 show the time evolution of the UV–vis spectra and hydrodynamic size of AgNPs in the cellular media of human cancer and fibroblast cells, respectively, highlighting the impact of BSA precoating on their stability. UV–vis absorbance spectra indicate that citrate-stabilized AgNPs tend to slightly aggregate in cellular medium, exhibiting a moderate peak broadening and the appearance of a shoulder in the absorption profile. However, they remain relatively stable over 16 h. In contrast, PAH-coated AgNPs undergo significant spectral changes, including the appearance of a second peak, indicative of extensive aggregation.^36^ The stability of AgNPs was drastically improved by precoating with BSA, particularly for PAH-coated ones, suggesting that the presence of BSA on the nanoparticle surface prevents uncontrolled agglomeration. These observations are corroborated by DLS measurements, where PAH-coated nanoparticles undergo a dramatic increase in size, exceeding 200 nm within 4 h, regardless of the type of cellular medium. After 16 h, they fully aggregate, making DLS measurements not possible. In contrast, PAH@BSA nanoparticles exhibit a much smaller size increase over time, demonstrating that BSA adsorption mitigates aggregation effects by providing steric stabilization.^34^ Similarly, cit@BSA nanoparticles remain more stable than their counterparts without BSA precoating, though both show only modest size increases. These results underscore the crucial role of the adsorption of protein in regulating nanoparticle stability in biological environments, particularly for positively charged coatings like PAH, which are otherwise prone to aggregation in cellular media.

**Figure 3:**
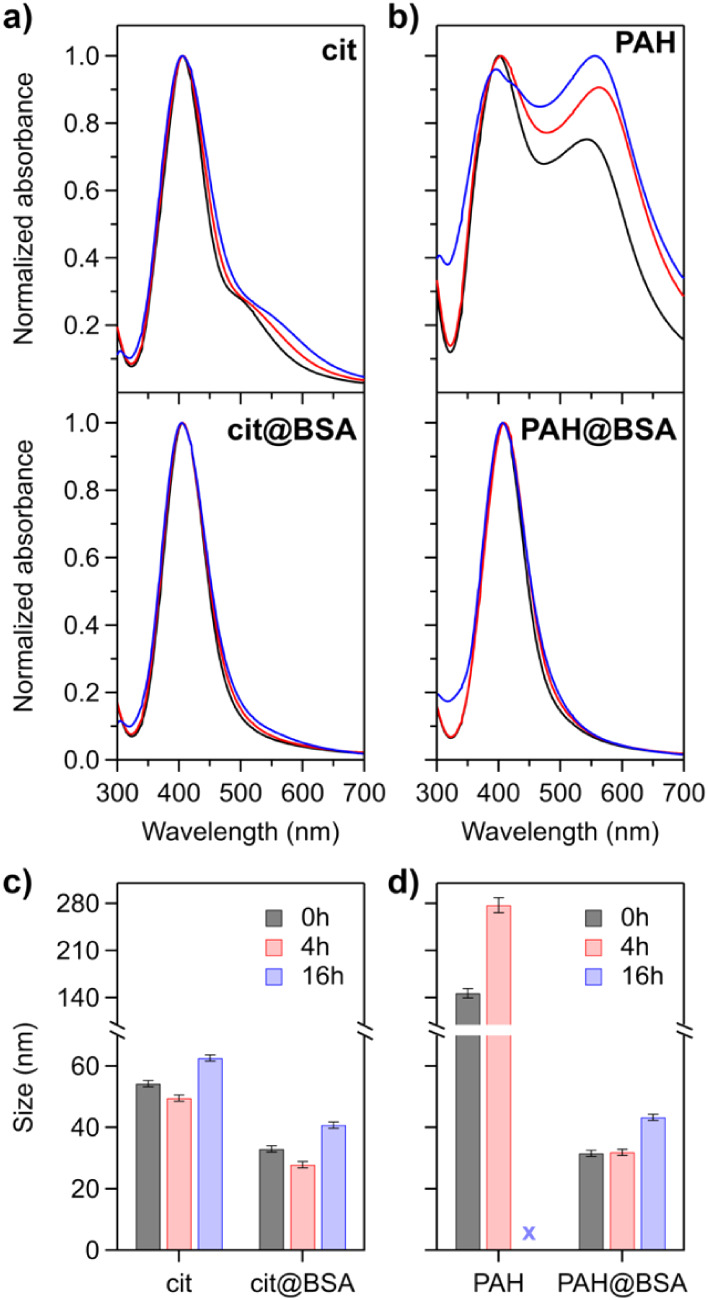
Optical and size characterization of AgNPs over time in human cancer cell line (MCF-7 and HT-29) medium. (a, b) UV–vis absorption spectra of AgNPs stabilized with citrate (cit) and poly­(allylamine hydrochloride) (PAH), with and without bovine serum albumin (BSA) precoating (cit@BSA and PAH@BSA). Spectra were recorded at different time points: 0 h (black), 4 h (red), and 16 h (blue). (c, d) Hydrodynamic size measurements of AgNPs-cit and AgNPs-PAH, with and without BSA precoating, over time (0 h, 4 h, and 16 h), as determined by dynamic light scattering (DLS).

To study the qualitative composition of the protein corona, the NP-protein complexes were separated by centrifugation, extensively washed, and the adsorbed biomolecules were isolated and analyzed by polyacrylamide gel electrophoresis (PAGE) coupled with protein staining (see Experimental Section). The gel shown in Figure 4a revealed a prominent protein band at approximately 66 kDa in all samples, confirming that the BSA is the major component forming the protein corona regardless of the precoating and the cellular media, as we have already observed.^33^ More importantly, both BSA-coated and uncoated NPs exhibited a highly similar band pattern, except for variations in band intensity reflecting differences in protein quantity, suggesting a qualitative comparable adsorption of serum proteins from the medium. However, a closer examination of the densitometric profiles (Figure 4b and c) reveals subtle differences between the different types of nanoparticles (i.e., citrate- and PAH-coated), while the BSA precoating appears to have a negligible impact. This suggests that the overall protein adsorption profile is primarily influenced by the intrinsic surface properties of the nanoparticles rather than by BSA precoating.

**Figure 4:**
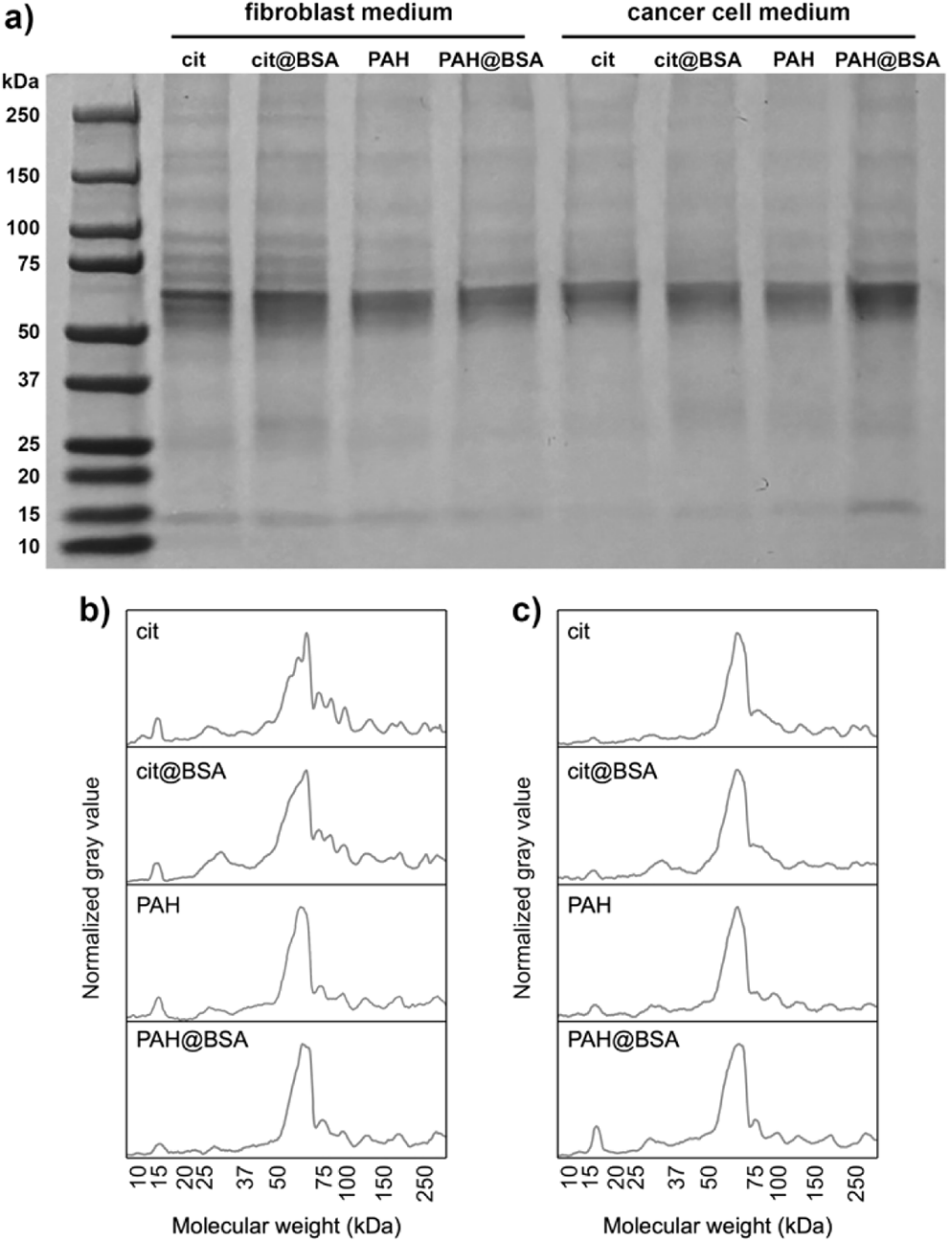
SDS-PAGE of biomolecules recovered from AgNPs after 24 h incubation with fibroblast (NIH-3T3) medium and cancer cell (MCF-7, HT-29) medium. (a) SDS-PAGE gel showing protein coronas formed on citrate- and PAH-coated AgNPs, with and without BSA precoating, after incubation in fibroblast medium (lanes 2–5) and cancer cell medium (lanes 6–9). The molecular weight ladder is shown in lane 1. Densitometric profiles of the SDS-PAGE lanes shown in panel (a), corresponding to AgNPs incubated with (b) fibroblast medium (lanes 2–5) and (c) cancer cell medium (lanes 6–9).

We then performed the BCA assay to evaluate whether the preadsorption of BSA onto AgNPs influences the total amount of proteins adsorbed when the nanoparticles are exposed to the cell culture medium. As shown in Figure S4, citrate-coated AgNPs tend to adsorb more proteins than PAH-coated ones, regardless of the cellular medium. This trend may be attributed to the higher tendency of PAH-coated nanoparticles to aggregate when exposed to the cell medium, as demonstrated by DLS measurements, which reduces the available surface area for protein adsorption. However, this difference between citrate-coated AgNPs and PAH-coated AgNPs remained consistent even after precoating with BSA, although this process significantly enhanced the stability of PAH-coated nanoparticles in the cell medium and reduced their tendency to aggregate. Therefore, the observed differences in protein adsorption between AgNPs-cit and AgNPs-PAH are most likely due to their distinct surface charges.

### 2.5 Serum-Free Toxicity of BSA-Precoated AgNPs

The above results confirm that BSA precoating contributes to nanoparticle stabilization but does not significantly influence either their cytotoxicity or the composition of the protein corona. To further explore whether BSA precoating directly affects the toxicity of AgNPs in the absence of a spontaneously formed protein corona, we assessed the cytotoxicity of BSA-coated AgNPs in MCF-7 and NIH-3T3 cells cultured in serum-free medium (i.e., without fetal bovine serum, FBS), where corona formation is prevented. In the absence of serum proteins, AgNPs-cit and AgNPs-PAH are unstable in saline and rapidly precipitate, forming a black aggregate, which precludes their use under these conditions.^34,35^ In contrast, as confirmed by UV–vis spectroscopy, BSA-coated nanoparticles remain stable when dispersed in serum-free medium, preserving their characteristic absorption profiles (Figure S5). As shown in Figure 5a, in the case of MCF-7 cells, after 24 h of incubation, cit@BSA nanoparticles are noncytotoxic even in the absence of serum proteins, similarly to what is observed in the presence of serum (Figure 2a). However, after 48 h, under serum-free conditions, they exhibit increased cytotoxicity compared to the serum-containing condition (Figure 2a). Likewise, PAH@BSA nanoparticles display cytotoxicity after 24 h that is comparable to that observed in the presence of serum proteins (Figure 2a), but show a higher cytotoxicity after 48 h (Figure 5a). As shown in Figure 5c,a similar trend is observed in NIH-3T3 cells. Under serum-free conditions,both cit@BSA and PAH@BSA-coated AgNPs induce cytotoxicity after 24 h comparable to that observed in the presence of serum proteins (Figure 2b), however after 48 h, cell viability is significantly reduced, indicating a stronger cytotoxic effect in the absence of serum. This increased cytotoxicity observed after 48 h of exposure under serum-free conditions, compared to normal conditions (i.e., in the presence of serum proteins), may be attributed both to the mitigating effect of the serum protein corona and to an increased cellular vulnerability caused by culturing in serum-free medium.

**Figure 5:**
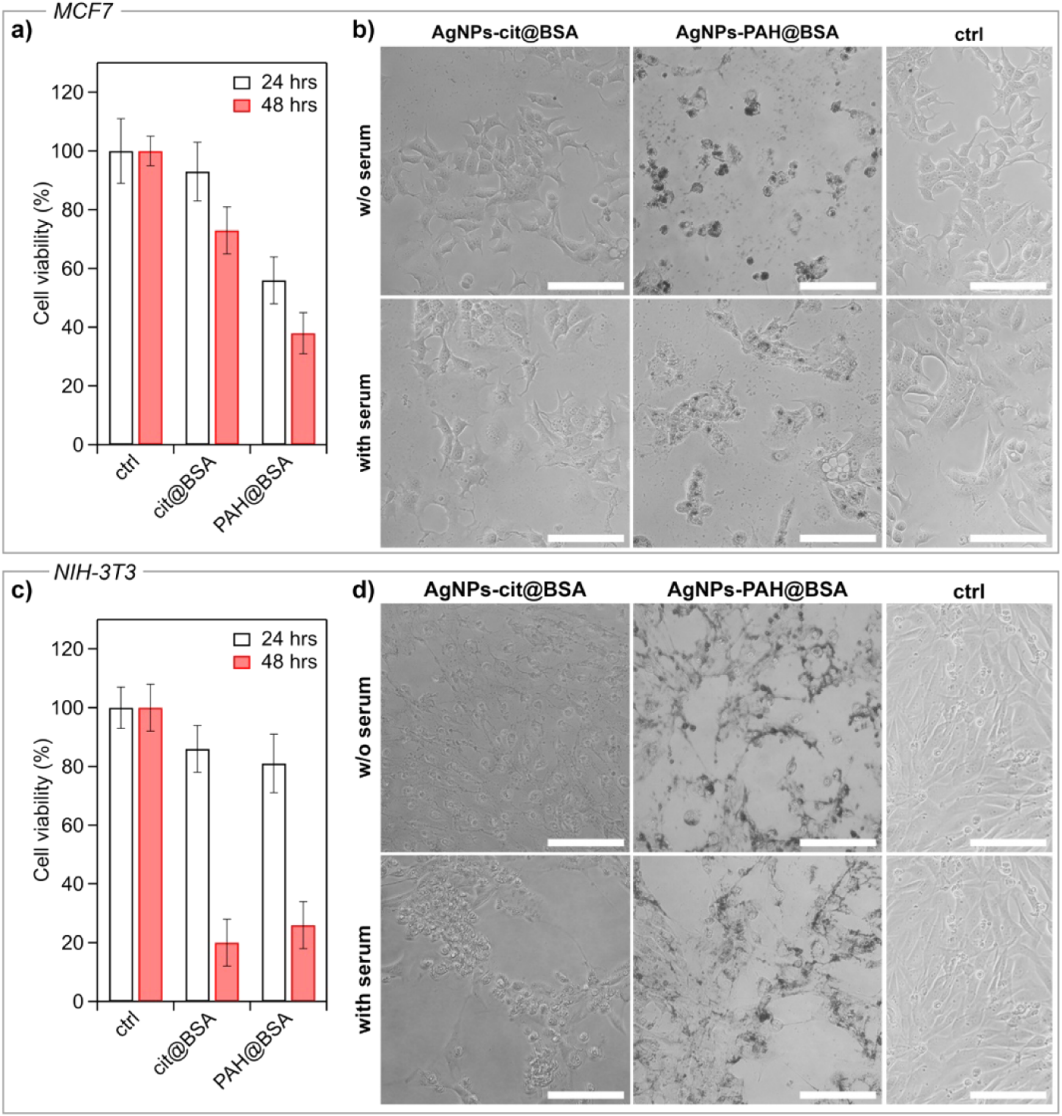
Serum-free cytotoxicity of BSA precoated AgNPs (20 μg/mL) in MCF-7 and NIH-3T3 cells. Cell viability of (a) MCF-7 and (c) NIH-3T3 cells after 24 and 48 h exposure to AgNPs-cit@BSA and AgNPs-PAH@BSA under serum-free conditions. Representative bright-field microscopy images of (b) MCF-7 and (d) NIH-3T3 cells incubated for 48 h with AgNPs-cit@BSA and AgNPs-PAH@BSA in the absence (w/o serum) and presence (with serum) of fetal bovine serum (FBS); untreated cells (ctrl) are shown for comparison. Data represent the mean ± SD and are expressed as a percentage relative to control samples (ctrl). At least three independent experiments were performed. Scale bars: 100 μm.

The bright-field microscopy images shown in Figure 5b,d qualitatively corroborate the viability data, providing morphological evidence consistent with the cytotoxicity trends observed under both serum-free and serum-containing conditions. In MCF-7 cells, only exposure to AgNPs–PAH@BSA induces pronounced morphological alterations, including cell rounding and loss of adhesion, consistent with the marked reduction in cell viability observed after 48 h, regardless of the presence of serum. Similarly, NIH-3T3 fibroblasts exhibit sensitivity to both AgNPs–cit@BSA and AgNPs–PAH@BSA, with severe morphological alterations observed after 48 h under both serum-free and serum-containing conditions. These include extensive cell detachment, loss of the characteristic elongated fibroblast morphology, and a pronounced reduction in cell density compared to the control, in agreement with the strong decrease in cell viability measured for both nanoparticles.

Overall, these results indicate that while BSA precoating ensures the colloidal stability of AgNPs under serum-free conditions, it does not override the intrinsic, surface charge– and cell type–dependent cytotoxic effects of the nanoparticles, which persist in both serum-containing and serum-free environments.

## 3 Conclusions

In this work, we examined how the formation of an artificial albumin corona influences the stability, protein adsorption, and biological activity of negatively and positively charged AgNPs. Our results demonstrate that BSA precoating markedly improves colloidal stability in biologically relevant media, particularly for positively charged PAH-coated nanoparticles, which are otherwise prone to rapid aggregation. Despite this stabilizing effect, BSA precoating does not substantially alter either the composition of the adsorbed proteins or the cytotoxic responses elicited by the AgNPs across the examined cell lines. Notably, under serum-free conditions, where spontaneous corona formation is prevented, BSA-coated AgNPs remain stable and biologically active, confirming that protein adsorption is essential for effective cellular interaction and uptake, while further demonstrating that surface charge ultimately governs the biological activity of AgNPs. Our findings indicate that, although the artificial albumin corona masks the surface charge of pristine AgNPs, the intrinsic surface charge of the nanoparticles remains dominant in dictating their toxicological profile. This raises important questions about which additional mechanisms contribute to these charge-dependent effects, including the possible involvement of the more dynamic and weakly bound layer of biomolecules, the so-called soft corona. Altogether, these insights underscore that both the spontaneous and artificial hard protein coronas are essential for stabilizing charged AgNPs in physiological environments and ensuring their bioavailability, while also highlighting the need to further investigate the contribution of the soft corona and other surface-dependent mechanisms to enable the rational design of engineered nanoparticles with predictable and tailored biological activity.

## 4 Experimental Section

### 4.1 Materials

Silver nitrate (AgNO_3_), sodium citrate (C_6_H_5_O_7_Na_3_), tannic acid (C_76_H_52_O_46_), poly­(allylamine hydrochloride) (PAH; MW = 17500 g/mol), MEM Non Essential Amino Acids (NEAA), Bovine Serum Albumin (BSA), Dulbecco’s phosphate buffered saline (DPBS) and 3-(4,5-dimethyl-2-thiazolyl)-2,5-diphenyltetrazolium bromide (MTT) were purchased from Merck and used without further purification. All aqueous solutions were prepared with deionized water obtained using an ultrafiltration system (Milli-Q, Millipore) with a measured resistivity above 18 MΩ·cm. Dulbecco’s modified Eagle medium (DMEM) and fetal bovine serum (FBS) were purchased from Gibco.

### 4.2 Synthesis of Citrate-Coated AgNPs (AgNps-cit)

AgNPs-cit were prepared following the method reported elsewhere.^33^ In brief, 100 mL of aqueous solution of sodium citrate (5 mM) and tannic acid (0.025 mM) was refluxed and an aqueous solution of silver nitrate (1 mL, 25 mM) was added quickly. Then, the reaction mixture was refluxed for 15 min, resulting in a bright yellow colloidal silver solution, and was then left to cool down to room temperature. The aqueous suspension of AgNPs was purified by two rounds of centrifugation (30000 g for 1 h at 6 °C) and resuspension in aqueous solution of sodium citrate (2 mM). The final concentration of AgNPs was calculated according to previously reported extinction coefficients.^37^

### 4.3 Positively Charged AgNPs (AgNps-PAH)

*AgNPs-PAH* were prepared as previously reported.^31^ In brief, the as-prepared AgNPs (1 mL) were transferred in deionized water (2 mL) and added dropwise to PAH (1 g/L, 2 mL) in NaCl (1 mM) aqueous solution under gently stirring. The excess of polymer was removed by two rounds of centrifugation (2000g for 1 h at 6 °C) and resuspension in deionized water.

### 4.4 Precoating with BSA of AgNps-cit

BSA solution (4.5 g/L,16.8 mL) in phosphate buffer (10 mM, pH = 7.4) were added to AgNPs-cit (1.2 mL) and left undisturbed overnight at room temperature. The excess of BSA was removed by two rounds of centrifugation (2000 g for 1 h at 6 °C) and resuspension in phosphate buffer (10 mM, pH = 7.4).

### 4.5 Precoating with BSA of AgNps-PAH

BSA solution (4.5 g/L,16.8 mL) in deionized water (pH < 4.7) were added to AgNPs-PAH (1.2 mL) and left undisturbed for 4 days at room temperature. The excess of BSA was removed by two rounds of centrifugation (2000 g for 1 h at 6 °C) and resuspension in phosphate buffer (10 mM, pH = 7.4).

### 4.6 Characterization of AgNPs

UV/vis spectra were recorded on a Jasco V-550 UV–vis-NIR spectrophotometer. Dynamic light scattering (DLS) and zeta potential measurement were performed in phosphate buffer (1 mM, pH = 7) and KCl (1 mM) or deionized water on a NanoBrook Omni Particle Size Analyzer (Brookhaven Instruments Corporation, USA) equipped with a 35 mW red diode laser (nominal 640 nm wavelength). AgNPs were characterized using Transmission Electron Microscopy (TEM) FEI Tecnai F20 ST equipped with a dispersion microanalysis of energy (EDS) and the Scanning Transmission Electron Microscopy (STEM) accessory. TEM samples were prepared by drop casting nanoparticle solutions onto a holey carbon-coated gold grid and dried at 80 °C. AgNPs coated with polyelectrolytes were stained with phosphotungstic acid (2 wt %, pH adjusted to 7 by adding NaOH) according to previously reported procedure.^38^ The average size and size distribution of citrate-stabilized AgNPs were measured by counting more than 800 particles. The TEM images were taken in the phase contrast mode and selected area electron diffraction (SAED). STEM pictures were recorded using High Angle Annular Dark Field (HAADF) detectors: in this imaging mode the intensity I is proportional to Z^1.7^t, where Z is the mean atomic number and t is the thickness of the specimen.

### 4.7 Serum Protein Adsorption

AgNPs were mixed in 1:14 volume ratio with complete medium (with or without 0.1 mM MEM NEAA) and incubated overnight at 37 °C. The nanoparticles were purified by four rounds of centrifugation (13000 g for 1 h at 6 °C) and resuspension in phosphate buffer (1 mM, pH = 7.4) and KCl (1 mM).

### 4.8 Quantification of Proteins

Proteins were quantified using the microbicinchoninic acid (micro-BCA) assay, as previously reported.^39^ AgNPs were transferred by centrifugation in 10% (w/v) SDS solution, warmed at 95 °C for 10 min, and then diluted with deionized water to a final SDS concentration of 5% (w/v). Nanoparticles were removed by centrifugation (13000 g for 1 h at 6 °C) and the supernatants were incubated with an equal volume of freshly prepared BCA working reagent at 60 °C for 1h. Absorbance at 562 nm was measured using a microplate reader (Thermo Scientific Varioskan Flash Multimode Reader) and total protein concentration calculated relative to the BSA standards.

### 4.9 Polyacrylamide Gel Electrophoresis (PAGE)

AgNPs were transferred by centrifugation in Tris-Cl (10 mM, pH 7.4), then resuspended in protein loading buffer (62.5 mM Tris-HCl pH 6.8, 10% (v/v) glycerol, 1% LDS, 0.0045% bromophenol blue, 50 mM DTT) and warmed at 95 °C for 5 min. Nanoparticles were removed by centrifugation (13000 g for 30 min) and the supernatants, along with a molecular weight ladder (Bio Rad), were loaded on 4–20% precast polyacrylamide gel (Bio-Rad) and resolved at 100 V for 60 min. The gels were stained with Coomassie Brilliant Blue G-250 for 1 h (0.2% w/v Coomassie Blue, 50% methanol, 10% glacial acetic acid) and subsequently destained for 3 h in 50% methanol and 10% acetic acid, followed by an overnight destaining step in 10% methanol and 10% acetic acid. Gel densitometry was performed using ImageJ 1.54g.

### 4.10 Cell Cultures

Human colon cancer cells, HT-29, and human breast cancer cells, MCF-7, were cultured under standard conditions in the DMEM medium supplemented with 10% (v/v) heat-inactivated FBS, 2 mM l-glutamine, 100 U mL^–1^ of penicillin and 100 U mL^–1^ of streptomycin in a humidified incubator set at 37 °C with 5% CO_2_. Mouse embryonic fibroblast cells, NIH-3T3, were cultured under standard conditions in the DMEM medium supplemented with 10% (v/v) heat-inactivated FBS, 2 mM l-glutamine, 0.1 mM MEM nonessential amino acids (NEAA), 100 U mL^–1^ of penicillin and 100 U mL^–1^ of streptomycin in a humidified incubator set at 37 °C with 5% CO_2_. Cells were seeded and grown for 24 h before exposure to nanoparticles. For experimental controls, the cell culture medium was diluted with deionized water or phosphate buffer (10 mM, pH = 7.4) (vehicle solution) to ensure that dilution of the medium by the solution of nanoparticles has no impact on the cell performance.

### 4.11 Actin and Nucleus Staining

Cells were seeded in 24-well plates and treated with AgNPs for 48 h. The cells were fixed with 4% paraformaldehyde in DPBS and washed with DPBS. They were then permeabilized with 0.001% Triton-X 100 (Merck Millipore). The cells were labeled with TRITC-conjugated phalloidin (FAK100, Merck Millipore) for 1 h, followed by rinses with DPBS. Nuclear counterstaining was performed by incubation with DAPI (4′,6-diamidino-2-phenylindole, Merck Millipore) for 3 min, followed by rinses with DPBS. The samples were examined using a Nikon Eclipse 80i microscope equipped for fluorescence analysis.

### 4.12 Cell Viability Assay

Cell viability was determined by MTT (3-(4,5-dimethylthiazol-2-yl)-2,5-diphenyltetrazolium bromide) assay measuring the intracellular reduction of tetrazolium salts into purple formazan by viable cells.^40^ Briefly, the cells were seeded in 96-well plates and treated with AgNPs for 24 or 48 h under standard conditions. After incubation, the medium with or without AgNPs was discarded, and the cells were washed two times with 100 μL of DPBS. Afterward, 100 μL of sterile MTT solution (1 mg mL^–1^ in DPBS) was added to each well and incubated for 1 h at 37 °C with 5% CO_2_. Subsequently, the medium was discarded and 200 μL of DMSO was added to each sample to solubilize formazan crystals. Optical density (OD) was read on a microplate reader (Thermo Scientific Varioskan Flash Multimode Reader) at 550 nm as a working wavelength and 640 nm as a reference. Cell viability was calculated as the proportion of the mean OD of the replicated wells relative to that of the control.

## Supplementary Material



## Notes

Additional cell viability data for HT-29 cells; fluorescence microscopy images of control and nanoparticle-treated MCF-7, HT-29, and NIH-3T3 cells; UV–vis spectra and DLS data showing time-dependent stability of AgNPs (cit, PAH, ± BSA) in different media; protein quantification by micro-BCA assay for all nanoparticle formulations; UV–vis spectra of BSA-coated AgNPs in serum-free conditions (PDF)
