# Supplementary material for: Impact of an Artificial Albumin Corona on Surface Charge-Driven Nano–Bio Interactions and Cytotoxicity of Silver Nanoparticles
Source: ACS Omega. 2026 Jun 15;11(25):37735–43. doi: 10.1021/acsomega.6c02733 (PMC13325155; doi:10.1021/acsomega.6c02733)
Supplement: Supplementary file 1 [file ao6c02733_si_001.pdf]

Supporting Information

Impact of an Artificial Albumin Corona on Surface Charge-Driven Nano-Bio Interactions and Cytotoxicity of Silver Nanoparticles

Marianna Barbalinardo,[a] Emilia Benvenuti, [a] Luana Mariani,[b] Andrea Migliori, [a] Lisa Lungaro,[c] Giacomo Caio, [c] and Denis Gentili,[*,a]*

a) Consiglio Nazionale delle Ricerche, Istituto per lo Studio dei Materiali Nanostrutturati (CNR-ISMN), via P. Gobetti 101, 40129 Bologna, Italy

b) Consiglio Nazionale delle Ricerche, Istituto per la Sintesi Organica e la Fotoreattività (CNR-ISOF), via P. Gobetti 101, 40129 Bologna, Italy

c) Department of Translational Medicine, St. Anna Hospital, University of Ferrara, 44124 Ferrara, Italy

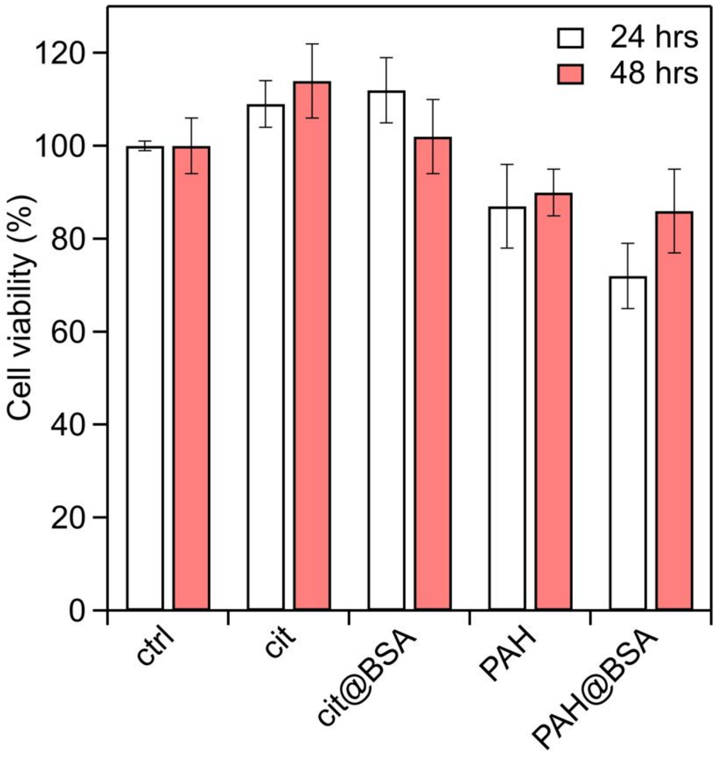

Figure S1. Cell viability of HT-29 cells treated for 24 and 48 hours with AgNPs (20 µg/mL) as a function of surface coating. Data represent the mean ± SD and are expressed as a percentage relative to control samples (ctrl). At least four independent experiments were conducted.

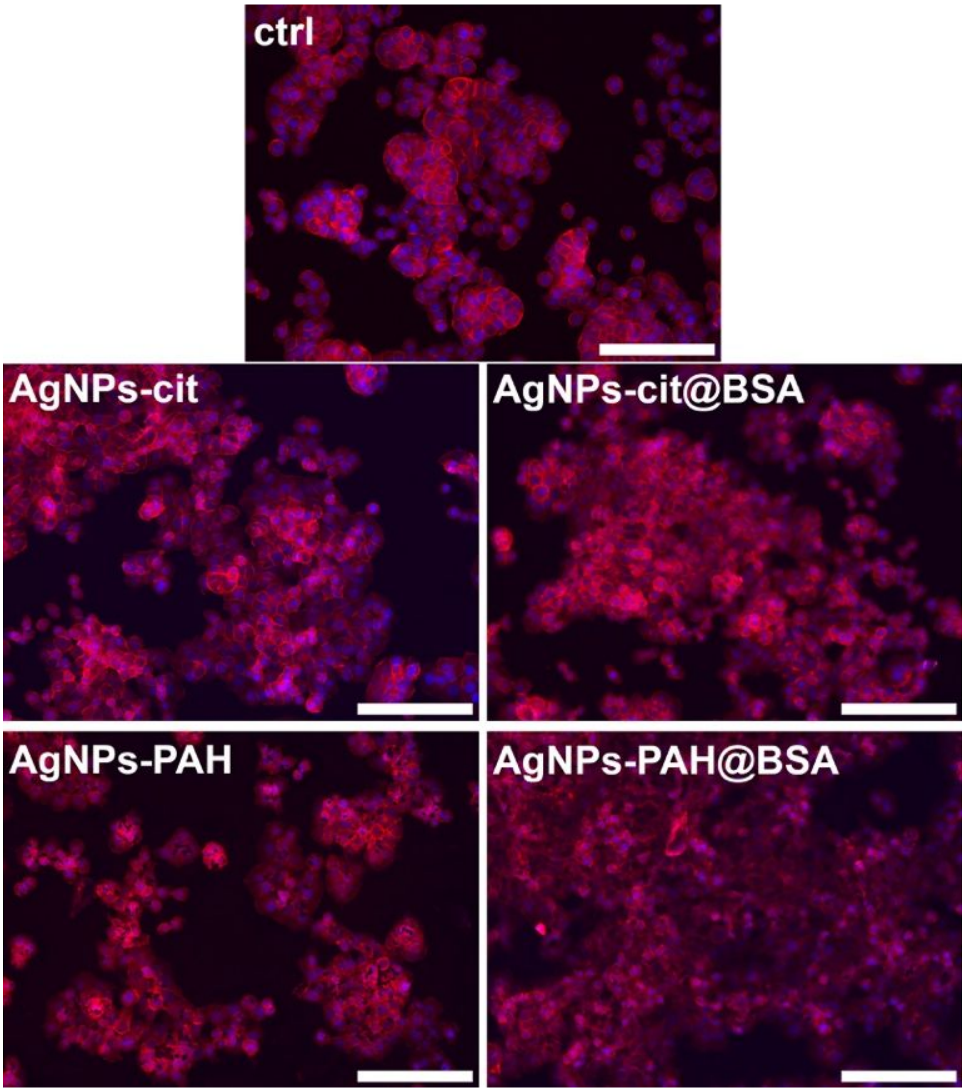

Figure S2. Fluorescence micrographs of HT-29 cells specifically labeled for actin (red) and nuclei (blue), treated for 48 hours with vehicle solution (ctrl), AgNPs-cit, AgNPs-cit@BSA, AgNPs-PAH and AgNPs-PAH@BSA (20 µg/mL). Scale bar: 100 µm.

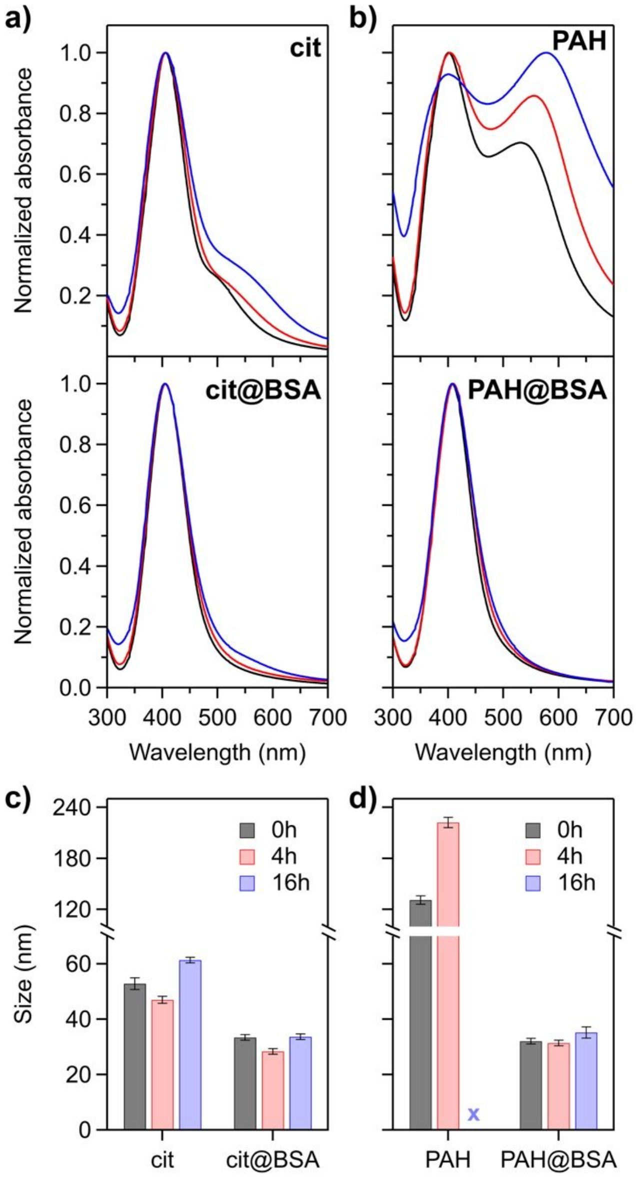

Figure S3. Optical and size characterization of AgNPs over time in fibroblast cell line (NIH-3T3) medium. (a, b) UV-Vis absorption spectra of AgNPs stabilized with citrate (cit) and poly(allylamine hydrochloride) (PAH), with and without bovine serum albumin (BSA) precoating (cit@BSA and PAH@BSA). Spectra were recorded at different time points: 0h (black), 4h (red), and 16h (blue). (c, d) Hydrodynamic size measurements of AgNPs-cit and AgNPs-PAH, with and without BSA precoating, over time (0h, 4h, and 16h), as determined by dynamic light scattering (DLS).

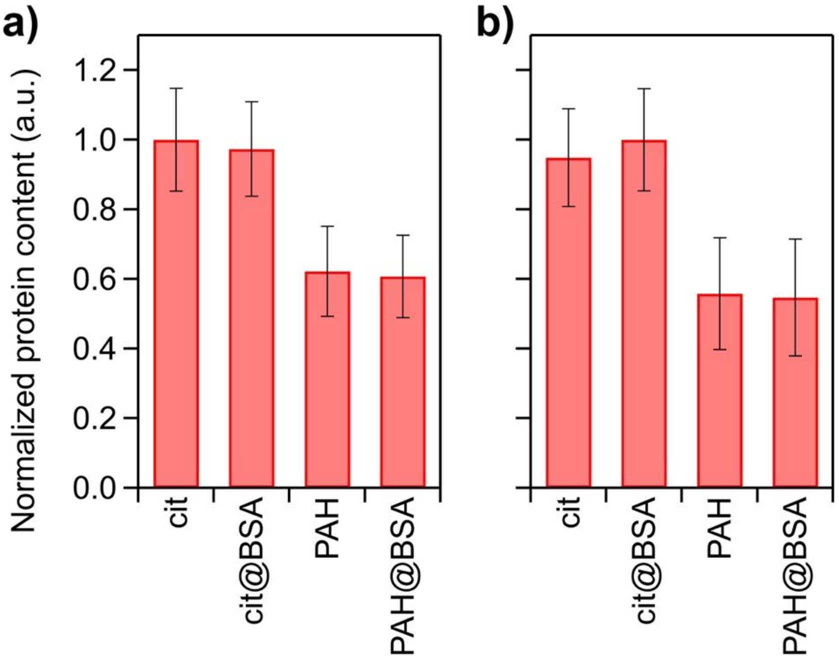

Figure S4. Normalized protein content determined by the BCA assay for AgNPs with different surface coatings after incubation in (a) MCF medium and (b) 3T3 medium. Error bars represent standard deviations from replicate measurements.

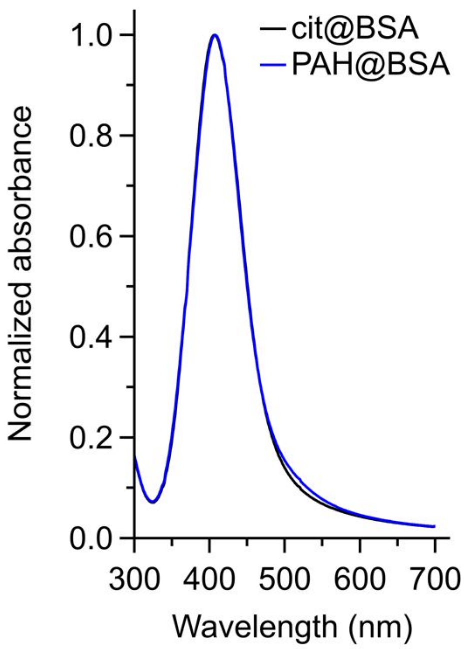

Figure S5. UV/v is absorption spectra of AgNPs-cit@BSA and AgNPs-PAH@BSA dispersed in serum-free medium.
